# Immunological and Clinical Effect of Diet Modulation of the Gut Microbiome in Multiple Sclerosis Patients: A Pilot Study

**DOI:** 10.3389/fimmu.2017.01391

**Published:** 2017-10-25

**Authors:** Marina Saresella, Laura Mendozzi, Valentina Rossi, Franca Mazzali, Federica Piancone, Francesca LaRosa, Ivana Marventano, Domenico Caputo, Giovanna E. Felis, Mario Clerici

**Affiliations:** ^1^Laboratory of Molecular Medicine and Biotechnology, Don Gnocchi Foundation, IRCCS, Milan, Italy; ^2^Department of Neurology, Don Gnocchi Foundation, IRCCS, Milan, Italy; ^3^Department of Biotechnology, University of Verona, Verona, Italy; ^4^Department of Physiopathology and Transplants, University of Milano, Milan, Italy

**Keywords:** multiple sclerosis, diet, microbiome, cytokine, inflammation, dysbiosis, immunology

## Abstract

Pathogenesis of autoimmune disorders, including multiple sclerosis (MS), has been linked to an alteration of the resident microbial commensal community and of the interplay between the microbiota and the immune system. Dietary components such as fiber, acting on microbiota composition, could, in principle, result in immune modulation and, thus, could be used to obtain beneficial outcomes for patients. We verified this hypothesis in a pilot study involving two groups of clinically similar relapsing-remitting (RR) MS patients who had undergone either a high-vegetable/low-protein diet (HV/LP diet group; *N* = 10) or a “Western Diet” (WD group; *N* = 10) for at least 12 months. Gut microbiota composition, analyzed by 16 S V4 rRNA gene sequencing and immunological profiles, was examined after a minimum of 12 months of diet. Results showed that, in the HV/LP diet group compared to the WD group: (1) *Lachnospiraceae* family was significantly more abundant; (2) IL-17-producing T CD4+ lymphocytes (*p* = 0.04) and PD-1 expressing T CD4+ lymphocytes (*p* = 0.0004) were significantly decreased; and (3) PD-L1 expressing monocytes (*p* = 0.009) were significantly increased. In the HV/LP diet group, positive correlations between *Lachnospiraceae* and both CD14+/IL-10+ and CD14+/TGFβ+monocytes (*R*_Sp_ = 0.707, *p* = 0.05, and *R*_Sp_ = 0.73, *p* = 0.04, respectively), as well as between *Lachnospiraceae* and CD4+/CD25+/FoxP3+ T lymphocytes (*R*_Sp_ = 0.68, *p* = 0.02) were observed. Evaluation of clinical parameters showed that in the HV/LP diet group alone the relapse rate during the 12 months follow-up period and the Expanded Disability Status Scale score at the end of the study period were significantly reduced. Diet modulates dysbiosis and improves clinical parameters in MS patients by increasing anti-inflammatory circuits. Because *Lachnospiraceae* favor Treg differentiation as well as TGFβ and IL-10 production this effect could be associated with an increase of these bacteria in the microbiota.

## Introduction

Multiple sclerosis (MS) is a chronic disease of the central nervous system (CNS) characterized by demyelination and mediated by an auto-reactive immune process directed against central neural tissues. Experimental autoimmune encephalitis (EAE) is a widely used animal model of MS induced by CNS-restricted antigens (1). The ethiopathogenesis of MS is still only partly understood, but a number of recent publications suggested that alterations of the microbiota play a role in the pathogenesis of this disease (2–4). Thus, the use of a cocktail of antibiotics to alter the gut microbiota of mice prior to EAE induction was shown to result in a significant reduction of EAE severity. This effect was mediated by an increase of CD4+/CD25+/FoxP3+ regulatory T cells (Treg) cells (5) and of regulatory CD5+/B cells (6). Even more recently, results indicated that EAE-resistant germ-free mice are rendered susceptible to the disease by the introduction of segmented filamentous bacteria into their gut microbiome. This phenomenon was the consequence of an increased differentiation of proinflammatory Th17 cells (7). Notably, dietary supplementation with probiotics was shown to modulate EAE secondarily to the regulation of pro- and anti-inflammatory cytokines (8–16), and engineered bacteria strains, such as *Salmonella*-CFA/I and Hsp65-producing *Lactococcus lactis*, were observed to prevent EAE *via* the production of TGFβ and IL-13 by Tregs (17–19). Finally, diet has been observed to influence EAE susceptibility and disease activity (2). Thus, a low-calorie diet was shown to have a beneficial effect in EAE (20), whereas a salt-rich diet resulted in an increased severity of EAE as a consequence of an upregulation of Th17 cell activity (21).

The analysis of gut microbiota in MS patients is still in the early stages (2). Recent data (22) showed that the human gut is colonized by *Clostridium perfringens* type B during disease relapse. *Clostridium perfringens* type B produces a toxin (ε toxin) that causes microangiopathy, resulting in the disruption of the blood–brain barrier (BBB) associated with neuronal and oligodendrocyte damage (23–26), possibly justifying its effect of disease activity. Additional data indicated that a mixture of *Clostridium* species enhances Treg cell populations in MS patients (27), suggesting that, besides the effect of a toxin, an imbalance within *Clostridium* species (phylum Firmicutes) might be present in the microbiota of these patients. Moreover, the archaeal Methanobrevibacteriaceae was described to be increased in MS patients, in whom the anti-inflammatory *taxa Butyricimonas* (phylum Bacteroidetes) and *Lachnospiraceae* (phylum Firmicutes) were decreased (28). Even more recently, *Faecalibacterium prausnitzii*, an important butyrate-producing organism, was observed to be reduced in MS patients in analogy to what is observed in inflammatory bowel disease, another autoimmune condition (29). Since butyrate upregulates Treg cell populations, these results suggest a mechanism by which gut microbiome alterations would predispose individuals to developing MS.

As diet plays an essential role in shaping the gut microbiome (30), and a high-fiber intake has been linked to health benefits *as a consequence of the effect of fiber* on the gut microbiota (31), possibly resulting in the modulation of the immune response, we hypothesized that MS disease activity could be affected by dietary patterns. In this pilot study, we verified this hypothesis by analyzing immune indexes, clinical parameters and gut microbiota in two groups of MS patients who at the time of recruitment were already following two distinct dietary regimes: a “Western Diet” (WD) and a high-vegetable/low-protein diet (HV/LP diet).

## Materials and Methods

### Individuals Enrolled in the Study

Patients with a diagnosis of relapsing-remitting (RR) MS that are followed by the Multiple Sclerosis Rehabilitation Unit of the Don Carlo Gnocchi Foundation in Milan, Italy, were enrolled in this pilot trial on a voluntary basis between May and October of 2016. Notably, this unit offers dietary advices to patients that are provided by a staff of professional nutritionists. Inclusion criteria were age >18 years and disease stability for >6 months prior to enrollment. Main exclusion criteria were: (1) use of disease modifying treatment (DMD) for >6 months prior to enrollment; (2) use of immunosuppressants or teriflunomide in the clinical history; (3) presence of significant co-morbidities such as arterial hypertension, cerebrovascular disorders, heart or pulmonary diseases, diabetes, endocrine, gastrointestinal, or psychiatric diseases. Gender, disease duration, and disability level, as assessed by the Kurtzke Expanded Disability Status Scale score (EDSS), relapse rate and other neurological indices were not used as inclusion/exclusion criteria but were recorded during the initial neurological examination.

Twenty-nine patients were initially selected for the study; all the patients underwent a face-to-face interview with a team of professional nutritionists who assessed the dietary regimen that had been followed for at least a 12-months period. This period was selected because it was considered to be a valid way to assess the adoption of a stable dietary habit. Nine of the initially selected patients were not enrolled in the study because adherence to clear dietary patterns could not be unequivocally identified. Of the remaining 20 patients, 10 (7 females and 3 males; median age = 43, IQ = 40–44) had chosen to follow a diet characterized by the use of fresh fruits and vegetables, legumes, nuts, whole grains, and extra virgin olive oil and a very limited use of animal proteins, including fish (no more than twice a week), poultry (no more than once a week), eggs (no more than four eggs a week), and dairy products (no more than once a week), as well as a low intake of refined cereals, salt, sugar, fried food and the exclusion of alcohol, red meat, saturated fats of animal origin, and trans-fats (e.g., processed dressing). This diet was labeled as high vegetable/low protein (HV/LP diet). The remaining 10 patients (8 females and 2 males; median age = 49, IQ = 45–52) were following a classical Western Diet (WD) characterized by the regular consumption of red meat, processed meat, refined grains, sweetened food, salt, and an overall high intake of saturated and omega-6 fatty acids (32). These 20 patients were enrolled in the study. Adherence to the two different dietary regimens was verified in a face-to-face interview with the professional nutritionists every 4 months. No use of any type of antibiotic or of pre- and probiotics was reported during the study period. Blood and fecal samples were collected at enrollment, i.e., after at least 1 year of either WD or HV/LP diet; neurological examinations were performed at enrollment and at the 12 months follow-up point.

The study protocol was approved by the ethics committee of the Don Carlo Gnocchi Foundation and all the enrolled patients signed an informed consent.

### Blood Sample Collection and Cell Separation

At enrollment whole blood (10 ml) was collected in vacutainer tubes containing ethylenediaminetetraacetic acid (EDTA) (Becton Dickinson & Co., Rutherford, NJ, USA). Peripheral blood mononuclear cells (PBMC) were separated on lympholyte separation medium (Cedarlane, Hornby, Ontario, CA, USA) and washed twice in PBS at 1500 RPM for 10 min; viable leukocytes were determined using a Scepter 2.0 Handheld Automated Cell Counter (Millipore, Billerica, MA, USA).

### Intracellular Cytokine or Transcription Factor Staining in PBMC

Lymphocyte and monocyte subsets were analyzed in freshly isolated PBMC that were incubated for 30 min at 4°C in the dark with Phycoerythrin-Cyanin-7 (PC7)-labeled anti**-**CD4 (clone SFCI12T4D11, mouseIgG_1_, Beckman-Coulter Brea, CA, USA), or PC7-labeled anti-CD14 (clone RMO52, mouse IgG_2a_, Beckman-Coulter), Phycoerythrin-Texas Red (ECD)-labeled anti-CD25 (clone B1.49.9, mouse IgG_2a_, Beckman-Coulter), Phycoerythrin (PE)-labeled anti-PD-1 (clone MIH4, mouse IgG1, eBioscience Cornerstone Court West, San Diego, CA, USA), PE-labeled anti-PD-L1 (clone MIH1, mouse IgG1, eBioscience), or PE-labeled anti-human Tim-3 (clone 344823, rat IgG_2A_, R&D Systems, Inc., Minneapolis, MN, USA). After incubation, the cells were washed, treated with Cell Permeabilization kit (FIX & PERM kit, eBioscience) and incubated for 30 min at 4°C in the dark with the following PE-labeled monoclonal antibodies: anti-IL-10 (clone JES9D7, mouse IgG1, R&D Systems), anti-TGFβ (clone 9016, mouse IgG1, R&D Systems), anti-IFNγ (clone 25723, mouse IgG_2b_, R&D Systems), anti-BDNF (clone 35909, mouse- IgG1, R&D Systems), anti-IL-25 (IL-17E, clone 182203, mouse IgG1, R&D Systems), anti-Gal-9 (clone 9M1-3, mouse IgG1_k_, Biolegend, San Diego, CA, USA), anti-RORCγ (clone AFKJS-9, rat IgG2a, eBioscience), anti-GATA-3 (cloneTWAY, rat IgG2B, eBioscience), anti-NFATc1 (clone H-10, mouse IgG_1_, Santa Cruz Biotechnology, Santa Cruz, CA, USA), or the Fluorescein Isothiocyanate (FITC)-labeled- anti-NFkB (clone C-5, mouse IgG_2a_, Santa Cruz Biotechnology), the PC-5-labeled-anti-IL-17 (clone BL168, mouse IgG_1k_, Biolegend), or the Alexa Fluor 488-labeled-anti-FoxP3 (clone 1054 C, rabbit IgG, R&D). Anti-Bat3 (clone 2B21, mouse IgM_k_, Abnova Taipei, Taiwan) was conjugated using the Lightning-LinkTM R-Phycoerythrin conjugation kit (Innova Biosciences, Cambridge, UK).

### Flow-Cytometry Analysis

Peripheral blood mononuclear cells were analyzed using a Beckman-Coulter GALLIOS flow cytometer equipped with a 22 mW Blue Solid State Diode laser operating at 488 nm and with a 25 mW Red Solid State Diode laser operating at 638 nm, and interfaced with Kaluza analysis software. Two hundred thousand cells were acquired and gated on lymphocyte and monocyte FSC and SSC properties. Isotype control or single fluorochrome-stained preparations were used for color compensation.

### Microbiome Analyses

At enrollment, participants were asked to collect their first morning stool at home using an adequate stool collection container (Biosigma, VE, ITALY). Samples were shipped, within 1 h, on ice packs to the Laboratory of the Don Carlo Gnocchi Foundation in Milan, where they were immediately stored at −80°C. Finally, the stored total stool samples were sent on dry ice by FedEx delivery to the processing facility (Second Genome Inc., San Francisco, CA, USA).

DNA isolation, library preparation, and sequencing as well as data analysis were performed by Second Genome Inc. Briefly, nucleic acid isolation with the MoBio PowerMag^®^ Microbiome kit (Carlsbad, CA, USA) and quantified *via* the Qubit^®^ Quant-iT dsDNA High Sensitivity Kit (Invitrogen, Life Technologies, Grand Island, NY). Samples enriched in bacterial 16 S V4 rDNA region and incorporating Illumina (San Diego, CA, USA) adapters and indexing barcodes, by PCR, were concentrated using a solid-phase reversible immobilization method for the purification of PCR products, quantified by qPCR and sequenced with MiSeq^®^ instrument. Amplicons were sequenced for 250 cycles with custom primers designed for paired-end sequencing. Operation taxonomic units (OTU) were selected using an in-house pipeline of analysis and sequences hitting a unique strain with an identity ≥99% were assigned a strain OTU. To ensure specificity of the strain hits, a difference of > = 0.25% between the identity of the best hit and the second best hit was required (e.g., 99.75 vs. 99.5); a chimera filtering and discard was also used. Representative OTU sequences were assigned taxonomic classification *via* mothur’s Bayesian classifier, trained against the Greengenes reference database of 16 S rRNA gene sequences clustered at 99%.

As for alpha-diversity (within sample diversity), observed diversity (number of unique OTU) and Shannon Index (which utilizes the richness of a sample along with the relative abundance of the present OTUs to calculate a diversity index) were the metrics used.

Sample-to-sample dissimilarity (beta diversity) was also determined. All profiles are inter-compared in a pair-wise fashion to determine a dissimilarity score and store it in a distance dissimilarity matrix. Distance functions produce low dissimilarity scores when comparing similar samples. Abundance-weighted sample pair-wise differences were calculated using the Bray–Curtis dissimilarity (ratio of the summed absolute differences in counts to the sum of abundances in the two samples) (33). The binary dissimilarity values were calculated with the Jaccard index (metric comparing the number of mismatches, i.e., OTUs present in one but absent in the other, in two samples relative to the number of OTUs present in at least one of the samples) (34).

Whole Microbiome Significance Testing was performed with Permutational Analysis of Variance (PERMANOVA), utilized for finding significant differences among discrete categorical or continuous variables. To identify differentially abundant taxa, a Wilcoxon Rank Sum test was employed. *p* values were adjusted by Benjamini–Hochberg procedure to control for false discovery rates from multiple testing. For additional information on laboratory methods and bioinformatic analyses please see Data Sheet S1 in Supplementary Material.

### Statistical Analysis

Quantitative data were not normally distributed (Shapiro–Wilk test) and are, thus, summarized as median and interquartile range (IQR; 25° and 75° percentile). Comparisons between two MS groups were made using a two-tailed Mann–Whitney *U* test performed for independent samples. The statistical correlations between immunological parameters and microbiota data were investigated by means of Spearman correlation coefficient and 95% confidence limits performed by Fisher’s *Z* transformation. The top 8 most abundant taxonomic families were compared by Kruskal–Wallis (KW) rank sum test. x^2^ test was used to patients relapse comparison. Statistical significance was set at a *p*-value <0.05. Data analysis was performed using the MedCalc statistical package (MedCalc Software bvba, Mariakerke, Belgium).

## Results

### Clinical Characteristics of the Individuals Enrolled in the Study

Demographic and clinical characteristics of the individuals enrolled in the study are summarized in Table 1. No differences were observed in gender, age, disease duration relapse rate, and EDSS score status when the two groups were compared at enrollment.

**Table 1:** Demographic and clinical characteristics of patients with a diagnosis of multiple sclerosis who were following either a Western Diet (WD) or a high-vegetable/low-protein (HV/LP) diet.

A	WD	HV/LP diet	*p*-Value
Number	10	10	
Gender (M:F)	2:8	3:7	
Age years (range years)	49 (45–52)	43 (40–44)	0.1
Disease duration years (range years)	12.5 (4.3–17.8)	8.8 (4–15)	0.4
Expanded Disability Status Scale (range)	2.0 (1.6–1.9)	1.8 (1.3–2.0)	0.3
Relapse rate (relapse number/disease years)	0.3 (0.3–0.8)	1 (0.0–1.0)	0.4

*Results obtained at enrollment (i.e., after 1 year of either WD or HV/LP diet) are shown. Data are reported as medians and interquartile range. Statistical significance is presented (*p* < 0.05)*.

### Diet-Associated Modifications of the Microbiota

Microbiota analyses were performed in all the MS individuals included in this study at enrollment, i.e., after at least 1 year of either HV/LP diet or WD. Sequences per sample ranged between 138,072 and 405,385 filtered reads and were sequenced to sufficient depth to capture OTU richness (Image 1). As a whole, 1,550 OTUs (combined filtered and strain level hits) were obtained from 8,852,375 combined sequences. All filtered reads were classified at the Kingdom level, 93.08% of reads were classified at the family level, 54.78% of reads were classified at the genus level, 22.7% of reads were classified at the species level and 23.87% of reads were classified at the strain level.

**Figure 1:**
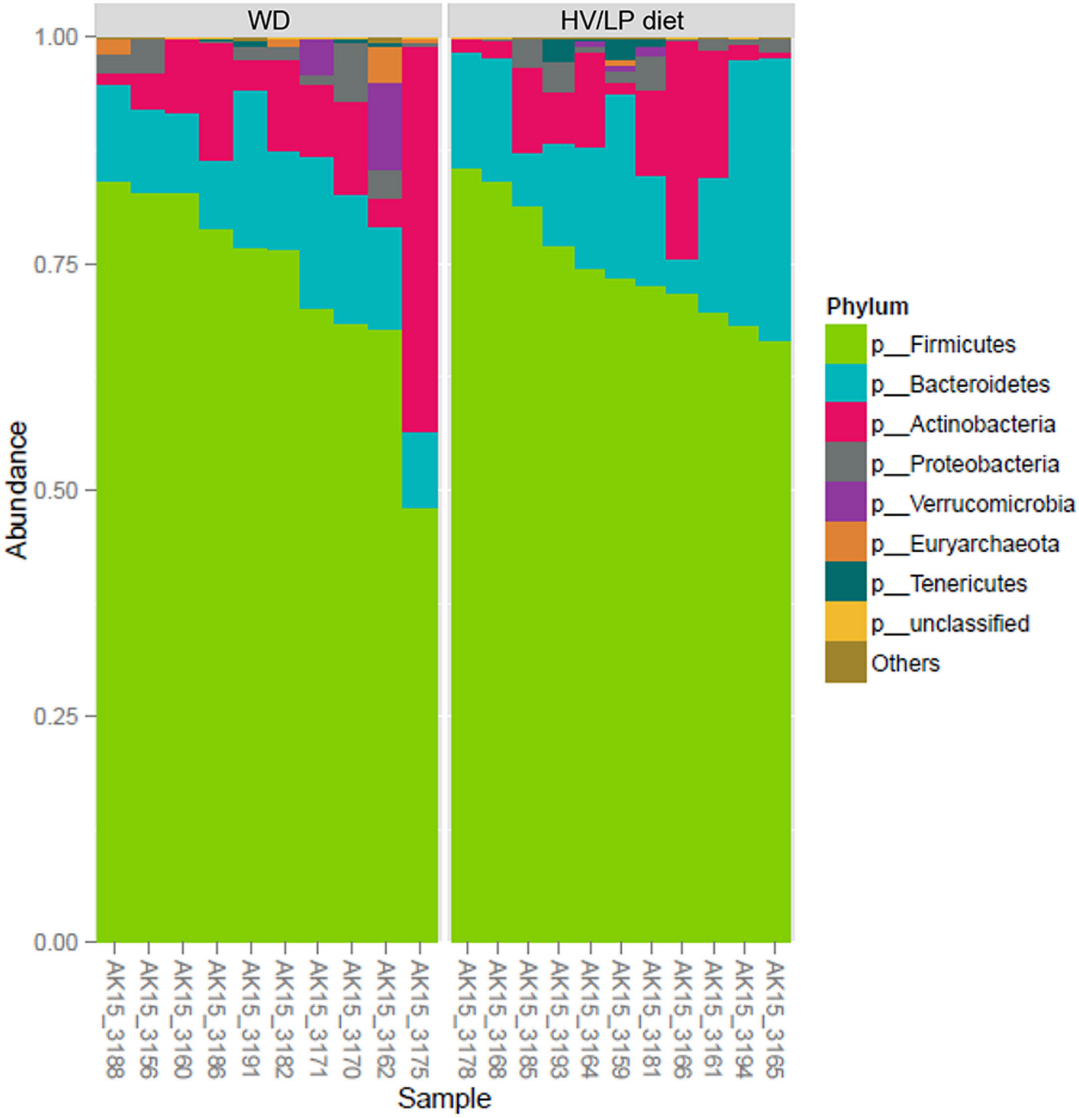
Firmicutes was the most abundant phylum in both groups (about 75%), followed by Bacteroidetes (15%), Actinobacteria (10%), and Proteobacteria (<2%). Plot showing the most abundant taxa at the Phylum level in multiple sclerosis (MS) patients who were following either a Western Diet (WD) or a high-vegetable/low-protein (HV/LP) diet. Stool samples were collected at enrollment, i.e., after at least 1 year of either WD or HV/LP diet.

**Figure 2:**
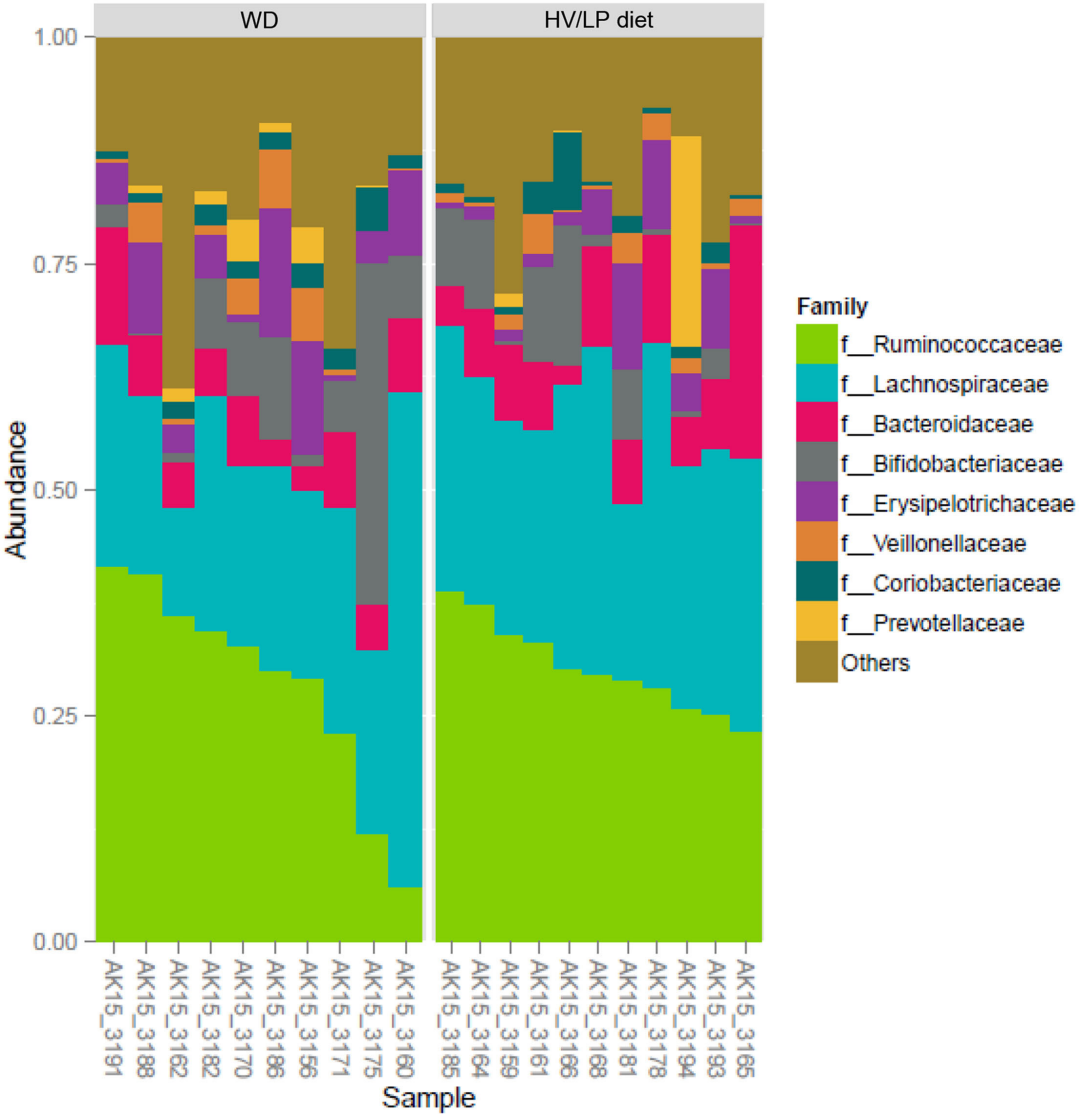
*Ruminococcaceae* (~30%) and *Lachnospiraceae* (~27%) were the most abundant Families, both belong to Firmicutes phylum. Plot showing the most abundant taxa at the Family level in multiple sclerosis patients who were following either a Western Diet (WD) or a high-vegetable/low-protein (HV/LP) diet. Stool samples were collected at enrollment, i.e., after at least 1 year of either WD or HV/LP diet.

**Figure 3:**
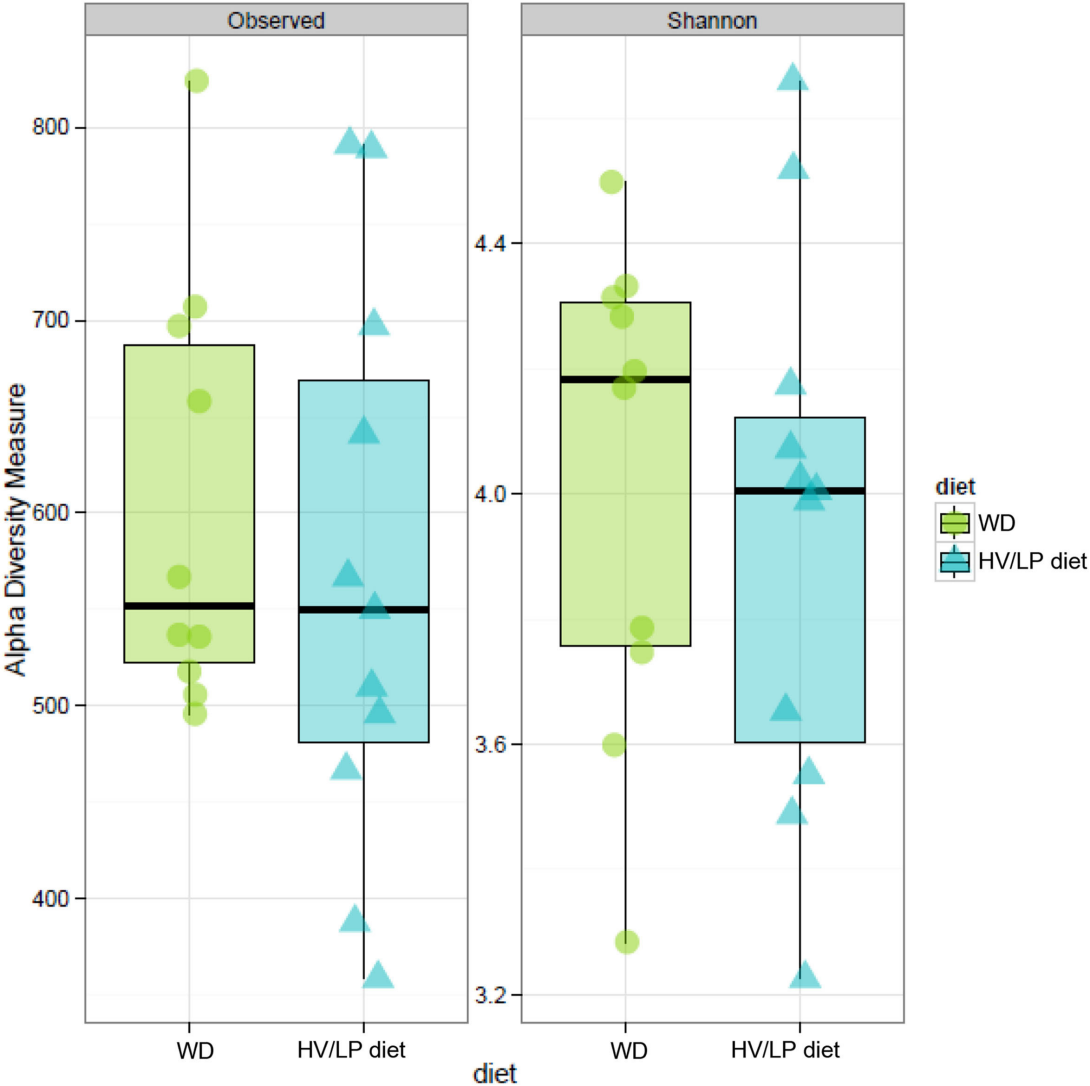
Estimates of alpha-diversity no shown significant difference. Observed diversity (left panel) represents the number of operation taxonomic unit (OTU) present in each sample, while Shannon diversity index (right panel) takes in account of richness and evenness of OTUs within a sample. Stool samples were collected at enrollment, i.e., after at least 1 year of either Western Diet or high-vegetable/low-protein diet.

Firmicutes was the most abundant phylum (Table 2; Figure 1) and *Ruminococcaceae* and *Lachnospiraceae* were the most abundant families in both groups of patients (Table 3; Figure 2). There was no difference in alpha-diversity when individuals following either one of the diets were compared (Figure 3), while *Lachnospiraceae* was significantly more abundant in the patients following HV/LP diet (Table 3) and phylum Euryarchaeota was significantly more abundant in WD patients (*p* = 0.03) (Table 2). No significantly different abundant OTUs between the two diets were observed at the time of sampling, even though 66 OTUs had an unadjusted *p*-value <0.05 and absolute log 2-fold change greater than 1 (data not shown). Moreover the taxa, namely *Coprococcus eutactus* (*p* = 0.3), *Ruminococcus lactaris* (*p* = 0.03) and a sequence of an as-yet unclassified *Lachnospiraceae* strain (*p* = 0.03), *Roseburia intestinalis* (*p* = 0.03), and a *Hungatella*-related unknown *Lachnospiraceae* member (*p* = 0.04) appeared to be more abundant in HV/LP (Table S1 in Supplementary Material).

**Table 2:** Median percent relative abundance and interquartile range of the most abundant taxa at the phylum level in patients with a diagnosis of multiple sclerosis who were following either a Western Diet (WD) or a high-vegetable/low-protein (HV/LP) diet.

Phylum	WD	HV/LP diet	*p*-Value	Chi-square
Firmicutes	76.8 (68.8–81.9)	73.5 (70.7–79.3)	0.9	2.7
Bacteroidetes	10.8 (8.9–13.7)	13.6 (11.8–17.6)	0.2	1.5
Actinobacteria	8.1 (3.6–10.2)	5.7 (1.6–9.9)	0.5	0.4
Proteobacteria	1.4 (0.5–3.0)	1.3 (0.4–2.3)	0.5	0.3
Verrucomicrobia	0.0 (0.0–0.04)	0.0 (0.0–0.3)	0.6	0.1
Euryarchaeota	0.04 (0.0–0.8)	0.0 (0.0–0.05)	**0.03**	4.3
Tenericutes	0.0 (0.0–0.4)	0.0 (0.0–0.5)	0.8	0.04
unclassified	0.1 (0.0–0.1)	0.05 (0.04–0.06)	0.8	0.03
Others	0.0 (0.0–0.1)	0.01 (0.0–0.03)	0.4	0.4

*Results obtained at enrollment (i.e., after 1 year of either WD or HV/LP diet) are shown. Statistical significance is presented (*p* < 0.05)*.

*The bold values are statistically significant p values*.

**Table 3:** Median percent relative abundance and interquartile range of the most abundant taxa at the family level in patients with a diagnosis of multiple sclerosis who were following either a Western Diet (WD) or a high-vegetable/low-protein (HV/LP) diet.

Family	WD	HV/LP diet	*p*-Value	Chi-square
*Ruminococcaceae*	31.5 (24.7–35.7)	29.7 (27.1–33.6)	0.83	0.04
*Lachnospiraceae*	21.8 (20.0–24.9)	29.5 (24.4–30.9)	**0.04**	**4.1**
*Bacteroidaceae*	6.0 (5.1–8.1)	7.7 (6.2–9.7)	0.36	0.8
*Bifidobacteriaceae*	6.2 (1.7–8.1)	3.5 (0.7–9.1)	0.67	0.1
*Erysipelotrichaceae*	4.8 (3.3–9.9)	1.6 (1.3–6.9)	0.32	0.9
*Veillonellaceae*	0.9 (0.5–4.3)	1.6 (0.6–2.2)	0.94	0.005
*Coriobacteriaceae*	1.9 (1.5–2.3)	1.0 (0.6–1.9)	0.11	2.6
*Prevotellaceae*	0.9 (0.0–1.4)	0.0 (0.0–0.1)	0.29	1.1
*Others*	16.6 (13.8–20.7)	16.1 (13.3–18.6)	0.48	0.4

*Results obtained at enrollment (i.e., after 1 year of either WD or HV/LP diet) are shown. Statistical significance is presented (*p* < 0.05)*.

*The bold values are statistically significant p values*.

Notably, although being suggestive of a role for the different dietary regimens in the changes in the microbiota composition, as baseline samples were not collected, it is not possible to definitely state that such changes are the direct consequence of the dietary regimens.

### Immune Parameters

**Figure 4:**
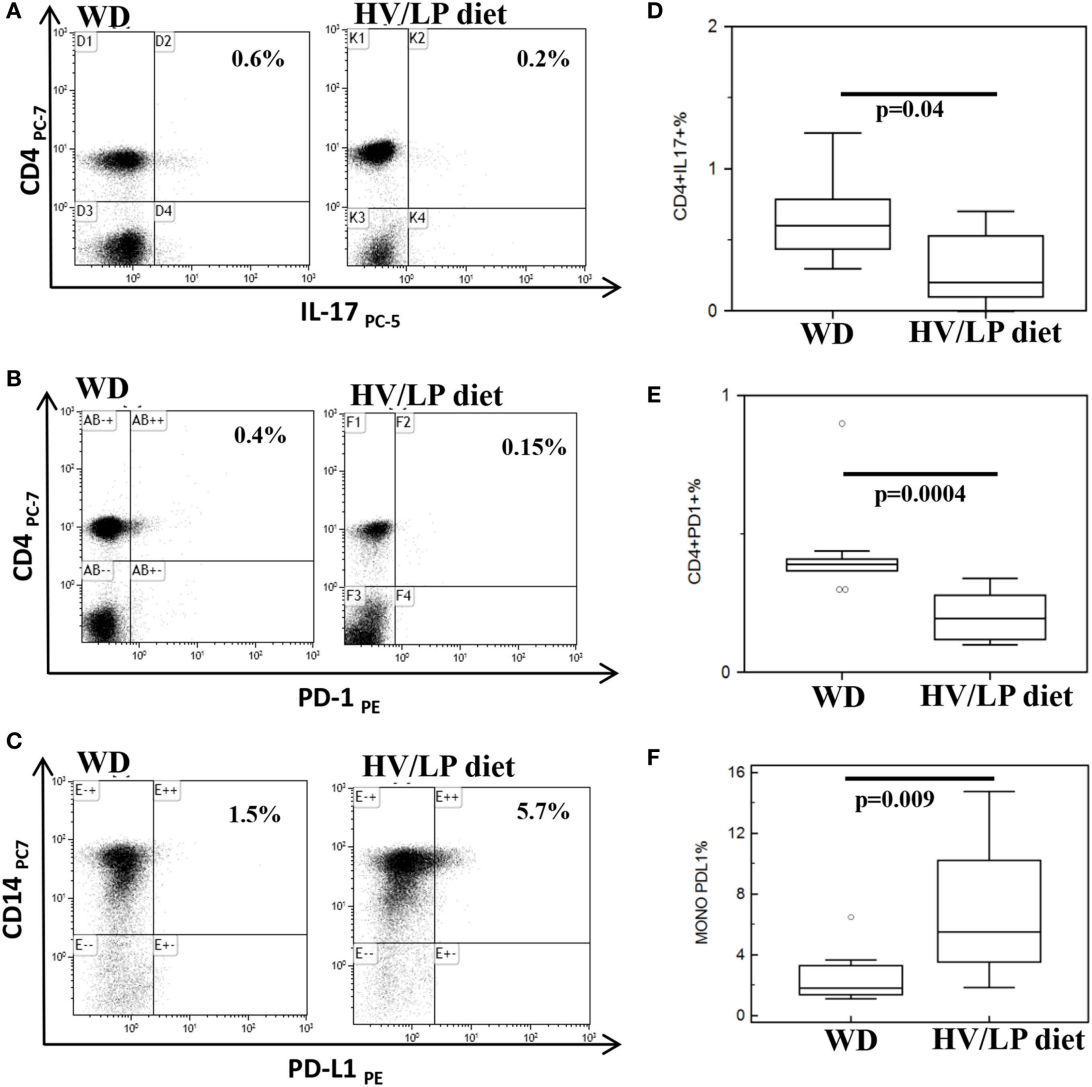
CD4+/IL-17+ and CD4+/PD-1+ T lymphocytes are decreased and CD14+/PD-L1+ cells are increased in multiple sclerosis (MS) patients following a high-vegetable/low-protein (HV/LP) diet. IL-17+/CD4+ T lymphocytes **(A)**; PD-1+/CD4+T lymphocytes **(B)**; and PD-L1+/CD14+ cells **(C)**. Representative results obtained in unstimulated peripheral blood mononuclear cell of MS patients who were following either a Western Diet (WD) or a HV/LP diet are shown. Top right corners show the percentage of CD4+/IL-17+, CD4+/PD-1+ T cells and of CD14+/PD-L1+ cells. Summary results are shown in **(D–F)**. The boxes stretch from the 25th to the 75th percentile; the lines across the boxes indicate the median values; the lines stretching from the boxes indicate extreme values. Statistical significance is shown. Blood samples were collected at enrollment, i.e., at least after 1 year of either WD or either HV/LP diet.

Immune parameters were analyzed in all the individuals at enrollment, i.e., after at least 1 year of either HV/LP diet or WD. Results showed that three cell populations were significantly different when HV/LP diet and WD were compared. Thus, in HV/LP diet compared to WD: (1) IL-17+/CD4+ as well as CD4+/PD-1+ T lymphocytes were reduced (*p* = 0.04 and *p* < 0.001, respectively) and (2) CD14+/PD-L1+ monocytes were augmented (*p* = 0.009) (Table 4; Figure 4). Finally, although not reaching statistical significance, possibly because of the small number of enrolled patients, a clear prevalence of anti-inflammatory monocytes was seen in the HV/LP diet individuals, in whom higher percentages of CD14+/TGFβ+ monocytes were detected (*p* = 0.09) (Table 4).

**Table 4:** Immune parameters in patients with a diagnosis of multiple sclerosis who were following either a Western Diet (WD) or a high-vegetable/low-protein (HV/LP) diet.

	WD	HV/LP diet	*p*-Value
CD4+CD25+FOXP3+	2.6 (2.0–3.0)	3.1 (2.3–3.6)	0.5
CD4+TIM-3+	0.8 (0.6–0.9)	1.0 (0.7–1.4)	0.3
CD4+GAL-9+	1.4 (0.7–1.7)	1.2 (0.6–2.0)	0.8
CD4+BAT3+	0.3 (0.2–0.3)	0.4 (0.3–0.4)	0.2
CD4+PD-1+	0.4 (0.4–0.4)	0.2 (0.1–0.3)	**0.0004**
CD4+NFATc+	0.2 (0.1–0.3)	0.1 (0.1–0.1)	0.07
CD4+NFkB+	0.2 (0.2–0.4)	0.2 (0.1–0.3)	0.6
CD4+GATA-3+	0.2 (0.1–0.4)	0.3 (0.2–0.4)	0.4
CD4+RORγ+	0.2 (0.1–0.3)	0.2 (0.2–0.3)	0.5
CD4+IL-10+	0.2 (0.1–0.2)	0.1 (0.1–0.2)	0.1
CD4+BDNF+	0.2 (0.1–0.2)	0.3 (0.2–0.3)	0.2
CD4+IL-25+	0.1 (0.0–0.2)	0.1 (0.0–0.2)	0.9
CD4+IL-17+	0.6 (0.5–0.7)	0.2 (0.1–0.5)	**0.02**
CD4+IFNγ+	0.5 (0.0–1.1)	0.4 (0.0–1.2)	0.7
CD14+IL-10+	0.5 (0.3–0.8)	0.9 (0.4–1.3)	0.6
CD14+TGFβ+	0.9 (0.7–1.2)	1.6 (0.9–1.9)	0.09
CD14+PD-L1+	1.8 (1.4–3.2)	5.5 (3.9–9.8)	**0.009**

*Results obtained at enrollment (i.e., after 1 year of either WD or HV/LP diet) are shown. Data are reported as medians and interquartile range. Statistical significances are presented (*p* < 0.05)*.

*The bold values are statistically significant p values*.

### Correlation between *Lachnospiraceae* and Anti-Inflammatory Immune Cell

*Lachnospiraceae*, the family of bacteria whose abundance was observed to characterize the microbiota of MS diet patients, were recently described to be associated with the preferential generation of an anti-inflammatory milieu. Thus, this family of bacteria facilitates Treg differentiation and stimulates TGFβ and IL-10 production by immune cells. To verify whether the *Lachnospiraceae* abundance seen in the HV/LP diet patients could be linked to a modulation of such cells, we analyzed possible correlations between *Lachnospiraceae* abundance and immune parameters. Results showed in HV/LP diet alone the presence of significantly positive correlations between *Lachnospiraceae* and both CD14+/IL-10+ and CD14+/TGFβ+ monocytes (*R*_Sp_ = 0.77, *p* = 0.008 and *R*_Sp_ = 0.73, *p* = 0.01; respectively), as well as between *Lachnospiraceae* and CD4+/CD25+/FoxP3+ T lymphocytes (*R*_Sp_ = 0.68, *p* = 0.02).

*Lachnospiraceae* were negatively correlated with both CD14+/IL-10+ and CD14+/TGFβ+ monocytes (*R*_Sp_ = −0.09, *p* = 0.8 and *R*_Sp_ = −0.48, *p* = 0.1, respectively) and positively correlated with CD4+/CD25+/FoxP3+ T lymphocytes (*R*_Sp_ = 0.02, *p* = 0.9) in WD; none of these correlations was statistically significant in this group of patients.

### Modulation of Disease Activity

Clinical parameters were evaluated during the 12 months follow-up period as well as at the end of the protocol; interesting differences emerged. Thus, whereas the EDSS score improved in the HV/LP diet patients, this parameter declined in WD patients, with a significant difference being observed between the two groups (*p* = 0.001). Notably, the overall number of disease relapses during the 12 months follow-up period was unmodified in the HV/LP diet patients, but increased significantly in the WD patients (vs. enrollment *p* = 0.04). Clinical relapses were observed during the 12 months follow-up period in 9/10 WD patients but only in 3/10 of the HV/LP diet patients (*p* = 0.0005); the difference between the two groups in relapse rate at the 12 months follow-up visit was statistically significant (*p* = 0.03). These results are shown in Table 5.

**Table 5:** Expanded Disability Status Scale (EDSS) scores, relapse rates, and number of patients in whom disease relapses were observed.

	WD	HV/LP diet	*P-*Value
EDSS (range) at baseline	2.0 (1.6–2.9)	1.8 (1.3–2.0)	0.44
EDSS (range) at the end of the follow-up period	2.5 (2.1–3.0)	1.0 (1.0–1.0)	**0.001**
Relapse rate (relapse number/disease years) at baseline	0.3 (0.3–0.8)	1.0 (0.0–1.0)	0.42
Relapse rate (relapse number/disease years) during the 12 months follow-up period	1.0 (1.0–1.0)	0.0 (0.0–1.0)	**0.03**
EDSS baseline vs. follow-up	0.31	0.06	
Relapse rate baseline vs. follow-up	**0.04**	0.6	

	**WD**	**HV/LP diet**	***P-*Value**

Patients in whom disease relapses were observed during the 12 months follow-up period	9/10	3/10	**0.0005**

*Two groups of multiple sclerosis patients following either a Western Diet (WD) or a high-vegetable/low-protein (HV/LP) diet were analyzed. Results obtained at enrollment (i.e., after 1 year of either WD or HV/LP diet) as well as during a 12 months follow-up period are presented. Medians, interquartile ranges and statistical significances are shown (*p* < 0.05)*.

*The bold values are statistically significant p values*.

## Discussion

Diet plays an essential role in shaping the composition of the gut microbiome (35), and the gut microbiota modulates the status of the immune response (36). In MS, in particular, changes in the composition of the microbiota were suggested to influence disease activity, and in the EAE murine model of MS tampering with microbiota can trigger or prevent disease development. To better define whether in MS different dietary regimens can modify the microbiota, if this results in a modulation of immune profiles, and, ultimately, whether diet-associated changes in the composition of the microbiota influence disease activity, we analyzed these parameters in two groups of MS patients who were following different diets. In particular, we compared microbiota composition and immune profiles in MS patients that had followed either a HV/LP or a WD diet for at least 1 year; clinical parameters were analyzed in these same individuals during a 12 months follow-up period. Results of this pilot study show that a skewing of the composition of the microbiota characterized by the abundance of *Lachnospiraceae* family, a decrease of IL-17-producing T CD4+ lymphocytes and PD-1 expressing T CD4+ lymphocytes, and an increase of PD-L1 expressing monocytes was observed in those individuals following a HV/LP diet. In these same patients, positive correlations between *Lachnospiraceae* and anti-inflammatory IL-10- and TGFβ-producing CD14+ monocytes, as well as between *Lachnospiraceae* and CD4+/CD25+/FoxP3+ Treg lymphocytes were also observed. Notably, a significant reduction of the EDSS score and of the relapse rate was observed during follow-up in the HV/LP diet group alone.

Different dietary regimens have convincingly been shown to influence the composition of the intestinal microbiota (35, 37, 38) and are suggested to modulate the clinical phenotype of a number of inflammatory and non-inflammatory conditions (39). Thus, whereas the microbiota was demonstrated to be different when breastfed and formula-fed neonates were compared, in adults dietary changes result in modifications of the gut microbiota. Recent results, in particular, indicated that a diet based on a high consumption of vegetables leads to an increase in the population of Firmicutes (*Roseburia, Ruminococcus bromii*, and *Eubacterium rectale*), whereas a primarily meat-based diet results in an increase in the abundance of bile-tolerant microbes (*Alistipes, Bilophila*, and *Bacteroides*) (35). We observed that the use of a HV/LP diet in MS patients was linked to an abundance of *Lachnospiraceae* bacteria in the gut microbiota. The *Lachnospiraceae* family belongs to the Firmicutes phylum, which are butyrate producers. This observation is important from an immunologic viewpoint, as butyrate is endowed with the ability to stimulate Treg activity and differentiation and to induce the generation of anti-inflammatory cytokines, including IL-10, by Treg cells (27, 40). Notably, data herein indicate that the abundance in *Lachnospiraceae* seen in the HV/LP diet MS patients was significantly correlated with increased percentages of peripheral Treg and of IL-10 and TGFβ-producing monocytes. In animal models, butyrate-producing bacteria were also shown to restore the integrity of the intestinal as well as of the BBB (41, 42), possibly reducing the translocation of peripheral blood inflammatory cells across the BBB.

We have previously shown that, whereas CD4+/Th17+ and CD4+/PD-1+ T lymphocytes are increased in MS compared to HC (43), CD14+/PD-L1+ monocytes prevail during disease remission (44); this is the immune profile we observed in MS patients undergoing a HV/LP diet. Taken together, these results could explain the attenuation of disease activity observed in these individuals during follow-up. Previous analyses of the microbiota composition in MS patients showed that *Methanobrevibacter*, bacteria that have been associated with inflammatory and autoimmune diseases (45), are increased in untreated MS (28) and this leads to a shorter relapse time (46). An increase of *Akkermansia* was also demonstrated in MS untreated patients (28, 47), in whom butyrate-producing *Faecalibacterium, Lachnospiraceae*, as well as *Ruminococcaceae, Bacteroides fragilis*, and *Butyricimonas* were reduced (28, 47, 48) Finally, *Prevotella*, a genus including many butyrate producers (49), was less abundant in MS untreated patients (4, 50), but an increase of this bacteria as well as of *Sutterella*, a species that drives anti-inflammatory response, was demonstrated in MS after treatment (28, 51). Results of this pilot study show a higher prevalence of *Lachnospiraceae* in MS patients following a HV/LP diet and are in accordance with previous data showing an increase of butyrate-producing bacteria in MS individuals after immunomodulatory treatment (28, 51). These data support the possibility that diet could be possibly be used as a tool to modulate the immune system in anti-inflammatory way as a consequence of changes in the gut microbiota (52, 53, 54). Notably, our results also showed that phylum Euryarchaeota was increased in WD patients alone. This finding could be of interest as this phylum was recently suggested to associate with shorter time to disease relapse in pediatric MS patients (55). Intriguingly, and underlining once again the intricacies of the interactions between the different components of the microbiota, a negative correlation between taxa belonging to family *Lachnospiraceae*, and Methanomassiliicoccales, which belong to phylum Euryarchaeota, was described (56). This is immunologically important as archaea of the Euryarchaeota phylum were suggested to interact with dendritic cells, resulting in the production of proinflammatory cytokines (57).

It is important to notice that this is a classic pilot study. Thus, because baseline samples were not collected, it is not possible to definitely state that the microbiota, immunologic, and clinical changes described herein are the direct consequence of the dietary regimens. These caveats notwithstanding, our data could be seen as supportive of the concept that diet-associated modifications of the composition of the microbiota modulate the immune response, and, in turn, this has an important impact on disease activity. It will be important to replicate these results in ampler cohorts of MS patients and to expand these observations in inflammatory diseases other that MS.

## Ethics Statement

All patients gave informed consent according to a protocol approved by the local ethics committee of the Don Gnocchi Foundation.

## Author Contributions

MS designed and performed the study and drafted the manuscript; LM designed the study and collected clinical data; FM organized patients enrollment and collected blood samples; DC selected patient groups and collected clinical data; GF organized and wrote the microbiota data; FP, FL, and IM performed immunological experiments and analyzed the results; VR conducted and analyzed nutritional interviews; MC coordinated the study and edited the manuscript. All authors reviewed and approved the final manuscript.

## Conflict of Interest Statement

The authors declare that the research was conducted in the absence of any commercial or financial relationships that could be construed as a potential conflict of interest.

## Abbreviations

MS, multiple sclerosis; CNS, central nervous system; EDSS, Expanded Disability Status Scale; IL, interleukin; OTU, operation taxonomic units; WD, Western Diet; HV/LP, high vegetal/low-protein DIET.
