# Supplementary figures and images for: Immunological and Clinical Effect of Diet Modulation of the Gut Microbiome in Multiple Sclerosis Patients: A Pilot Study
Source: Front Immunol. 2017 Oct 25;8:1391. doi: 10.3389/fimmu.2017.01391 (PMC5661395; doi:10.3389/fimmu.2017.01391)

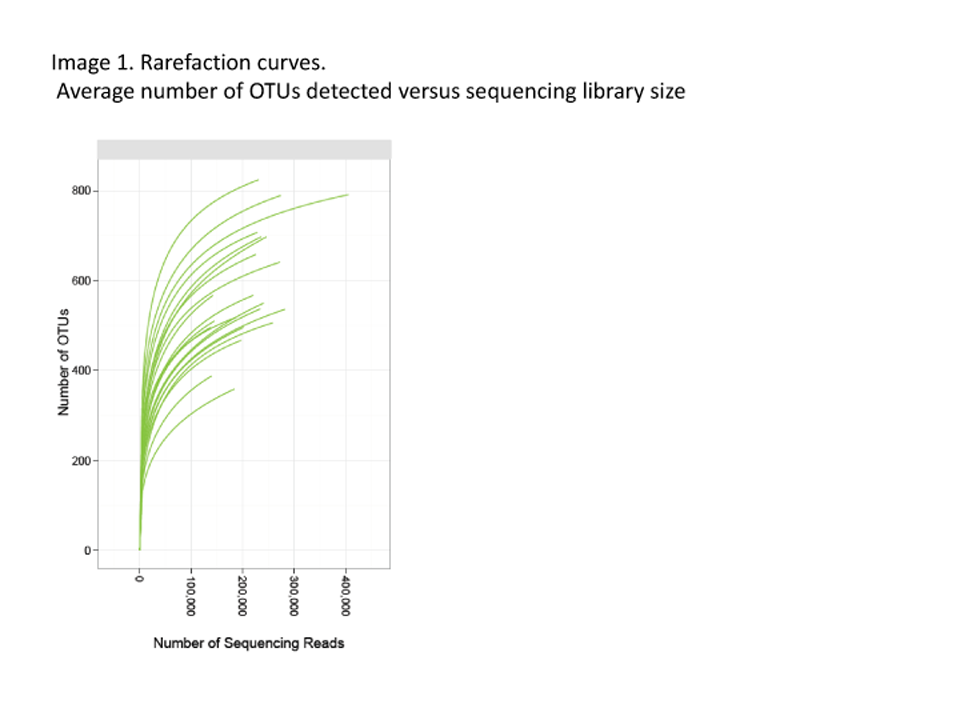

Supplement: Supplementary file 1 [file image_1.tif]
