# Supplementary material for: Immunological and Clinical Effect of Diet Modulation of the Gut Microbiome in Multiple Sclerosis Patients: A Pilot Study
Source: Front Immunol. 2017 Oct 25;8:1391. doi: 10.3389/fimmu.2017.01391 (PMC5661395; doi:10.3389/fimmu.2017.01391)
Supplement: Supplementary file 2 [file table_1.docx]

**Supplementary Table S1.** OTUs of *Lachnospiraceae* family which relative abundance median is higher in HV/LP diet, ordered according to the ratio of median in HV/LP diet vs. WD. *Data are reported as median values and interquartile range. Statistical significances are presented (p<0.05).*

| **ID** | **OTU** | **Genus** | **Species** | **Strain/Best hit** | **Percent Median**  **WD** | **Percent Median**  **HV/LP diet** | **Ratio**  **percent median**  **MS diet vs**  **MS control** | **p value** |
| --- | --- | --- | --- | --- | --- | --- | --- | --- |
| 1352 | t__38993 | g__Coprococcus | s__eutactus | *Coprococcus eutactus* ATCC 27759 | 0.02  (0.02-1.12) | 1.10  (0.02-15.7) | 60.89 | 0.3 |
| 1318 | t__27728 | g__94otu9710 | s__97otu18534 | *Ruminococcus lactaris* ATCC 29176 | 0.01  (0.0-0.04) | 0.29  (0.06-0.90) | 49.83 | **0.03** |
| 173 | dnOTU_117 | g__unclassified | s__unclassified | t__unclassified  *Ruminococcus* g5 (99,60 % identity)  *Clostridium* *glycyrrhizinilyticum* (98.8%) | 0.01  (0.0-0.16) | 0.42  (0.06-0.65) | 48.24 | **0.03** |
| 873 | dnOTU_64 | g__94otu14231 | s__97otu397 | t__unclassified *Clostridium* g8 (100%) | 0.01  (0.0-0.20) | 0.11  (0.0-1.0) | 15.47 | 0.7 |
| 1317 | t__27000 | g__Roseburia | s__97otu25366 | *Roseburia intestinalis* L1-82 | 0.04  (0.01-0.20) | 0.47  (0.30-1.70) | 11.34 | **0.03** |
| 857 | dnOTU_625 | g__Blautia | s__unclassified | t__unclassified  Candidatus *Blautia* PAC0007575 (97,78%)  *Blautia faecis* M7-8 (Candidatus) and M25T (96,81%) | 0.06  (0.01-0.38) | 0.57  (0.13—1.70) | 9.46 | 0.07 |
| 459 | dnOTU_267 | g__Lachnospira | s__unclassified | t__unclassified  *Eubacterium eligens* (98,41%) | 0.08  (0.03-0.26) | 0.46  (0.03-0.63) | 5.60 | 0.2 |
| 335 | dnOTU_155 | g__94otu11726 | s__97otu97516 | t__unclassified  unknown Lachnospiraceae 100%  *Hungatella* (98.8%) | 0.05  (0.03-0.15) | 0.30  (0.20-0.58) | 5.60 | **0.04** |
| 1300 | t__22024 | g__Lachnospira | s__97otu55 | *Eubacterium eligens* ATCC 27750 | 0.44  (0.08-1.75) | 2.00  (0.14-2.95) | 4.53 | 0.2 |
