# Supplementary material for: Immunological and Clinical Effect of Diet Modulation of the Gut Microbiome in Multiple Sclerosis Patients: A Pilot Study
Source: Front Immunol. 2017 Oct 25;8:1391. doi: 10.3389/fimmu.2017.01391 (PMC5661395; doi:10.3389/fimmu.2017.01391)
Supplement: Supplementary file 3 [file data_sheet_1.docx]

Supplementary methods

*Laboratory methods*

Stool sample sequencing and analyses were performed by Second Genome, Inc., San Francisco, CA. Specifically, nucleic acid isolation was performed using the MoBio PowerMag® Microbiome kit (Carlsbad, CA) optimized on the ThermoFisher KingFisher Nucleic Acid Purification System for high-throughput processing. All samples were quantified via the Qubit® Quant-iT dsDNA High Sensitivity Kit (Invitrogen, Life Technologies, Grand Island, NY) to ensure that they met minimum concentration and mass of DNA. To enrich the sample for bacterial 16S V4 rDNA region, DNA was amplified with 515F-806B primers that were tailed with sequences to incorporate Illumina® (San Diego, CA) adapters and indexing barcodes. Each PCR product was quantified by fluorometric method (Qubit or PicoGreen from Invitrogen, Life Technologies, Grand Island, NY), and pooled equimolar for sequencing. A pooled library was loaded onto the Illumina MiSeq® platform. After cluster formation on the MiSeq instrument, the amplicons were sequenced for 250 bp paired-end sequencing.

*Operational taxonomic unit (OTU) selection*

Sequenced paired-end reads were merged using USEARCH (1) and the resulting sequences were compared to an in-house strains database using USEARCH. All sequences matching to a unique strain with an identity ≥99% were assigned a strain OTU. To ensure specificity of the strain hits, a difference of ≥0.25% between the identity of the best hit and the second best hit was required (e.g., 99.75 versus 99.5). For each strain OTU, one of the matching reads was selected as representative and all sequences were mapped by USEARCH against the strain OTU representatives to calculate strain abundances. The remaining non-strain sequences were quality filtered and de-replicated with USEARCH. Resulting unique sequences were then clustered at 97% by UPARSE (1) (de novo OTU clustering) and a representative consensus sequence per de novo OTU was determined. The UPARSE clustering algorithm includes a chimera filtering and discards likely chimeric OTUs. All non-strain sequences that passed the quality filtering were mapped to the representative consensus sequences to generate an abundance table for de novo OTUs. Representative OTU sequences were assigned taxonomic classification via the mothur’s (2) bayesian classifier trained against the Greengenes (3) reference database of 16S rRNA gene sequences clustered at 99%.

1. Edgar RC. Search and clustering orders of magnitude faster than BLAST. *Bioinformatics* (2010) **26**(19): 2460-2461.
2. Schloss PD, Westcott SL, Ryabin T, Hall JR, Hartmann M, Hollister EB,et al. Introducing mothur: Open-source, platform-independent, community-supported software for describing and comparing microbial communities. *Appl Environ Microbiol* (2009) **75**(23):7537-41.
3. McDonald D, Price MN, Goodrich J, Nawrocki EP, DeSantis TZ, Probst A, et al. An improved Greengenes taxonomy with explicit ranks for ecological and evolutionary analyses of bacteria and archaea. *ISME* *J* (2012) **6**: 610–618.
